# An Unexpected Application of the Negative Root Arising from the Use of the Systematic Treatment of Equilibrium in Calculating pH Contribution from the Autoprotolysis of Water in Highly Diluted Strong Acid or Base Solutions

**DOI:** 10.1021/acsomega.6c07168

**Published:** 2026-09-29

**Authors:** Gabriel Unger, Chong Qiu

**Affiliations:** Department of Chemistry and Chemical & Biomedical Engineering, Tagliatella College of Engineering, 8518 University of New Haven, 300 Boston Post Road, West Haven, Connecticut 06516, United States

## Abstract

In the undergraduate analytical chemistry curriculum, determining the concentration of hydronium ions (H^+^) of a highly diluted strong acid or base solution (below 10^–6^ M) is an important example to demonstrate the concept of the systematic treatment of chemical equilibria using applicable charge balance, mass balance, and chemical equilibrium constants. In this case, to accurately describe the pH of the solution, the autoprotolysis of water must be considered in addition to the dissociation/protonation of the strong acid/base. Such systematic treatment yields a quadratic equation with [H^+^], the concentration of H^+^, as its unknown. Solving the quadratic equation always yields a positive root equaling the total [H^+^] of the solution and a negative one with no significant value. This article shows, based on the mass balance equations in such a chemical system, that the absolute value of this negative root is the [H^+^] from the autoprotolysis of water in the system. Our findings could be an interesting critical thinking activity in undergraduate curricula to assist students in their understanding of the principle and application of the systematic treatment of equilibria and practice critical thinking.

## Graphical abstract

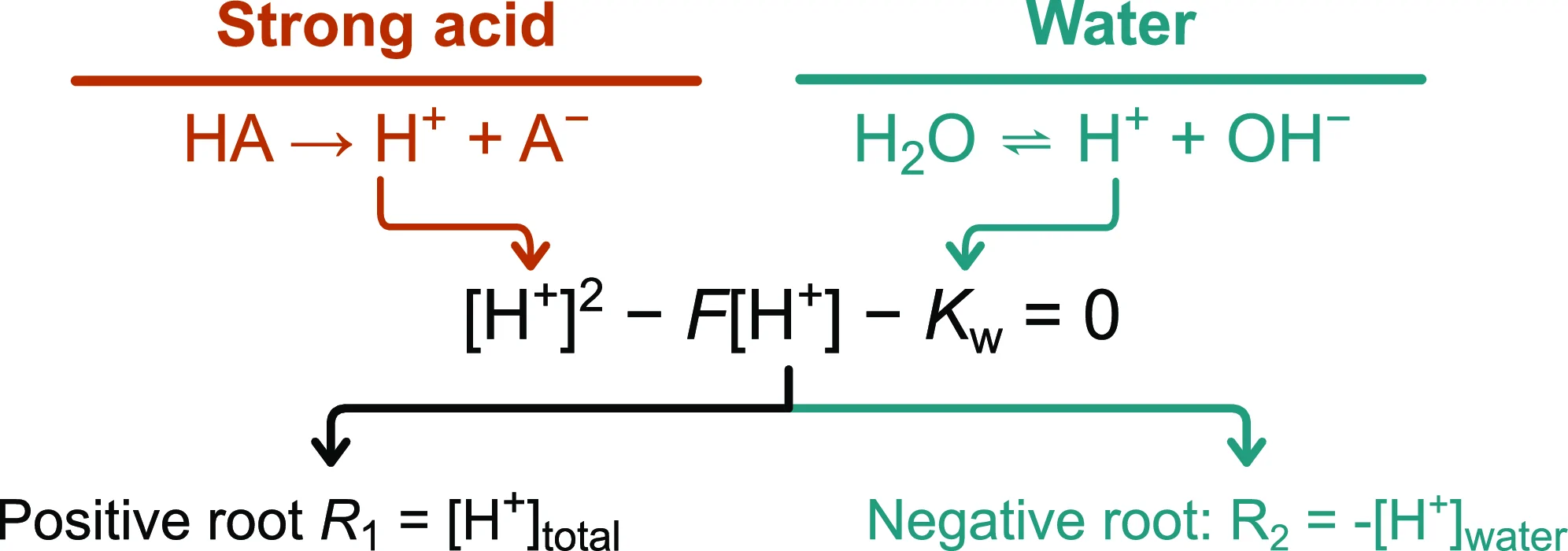

## Introduction

An important topic in the undergraduate analytical chemistry curriculum is the systematic treatment of chemical equilibria in aqueous systems. This topic is normally discussed in quantitative analysis courses designed for second-year undergraduate students, typically through examples of pH calculations of aqueous solutions.^1–3^ The pH of an aqueous solution is of particular interest to the science and engineering communities because of its importance in buffer solutions, human physiology, pharmacology, and manufacturing and environmental processes. Therefore, pH calculations hold a critical place in undergraduate chemistry curricula.

The pH of an aqueous solution is defined as the negative log of the thermodynamic activity of all hydronium ions, H_3_O^+^ (noted as H^+^ for compactness), in such a chemical system. A complex challenge is the pH calculation of an aqueous solution with two or more acid–base equilibria. In this case, the concentration of H^+^ (denoted as [H^+^] throughout this article) can no longer be solved by using just any one of the reactions involved. Instead, a series of equations based on the charge balance, mass balance, and related chemical equilibrium expressions should be established to solve for the total [H^+^] and convert it to the proper thermodynamic activity for pH determination.

## Results

### Systematic Treatment of a Highly Diluted Strong Acid Solution

A classic classroom example of the systematic treatment of chemical equilibria is the pH calculation of a highly diluted strong acid or base solution. In undergraduate classes, two approximations are commonly made: (1) the total ion concentration in the system is low (e.g., 0.1 mM or less) so that the activity coefficients approach unity and thermodynamic activities are approximately the same as the concentrations of the involved ions;^1^ and (2) such a chemical system is closed with no exposure to CO_2_. These approximations help the students focus on the new concept of systematic treatment of chemical equilibria. These two assumptions are used throughout the rest of this article.

Normally, the pH of a strong acid solution with a reasonably high concentration is calculated by assuming that the acid is the sole source of H^+^ in the solution while assuming the autoprotolysis of water is negligible. Such approximation, valid when the formal concentration of the acid is sufficiently high, offers a quick way to estimate the solution pH by calculating the negative log of [H^+^] (equals the formal concentration *F* of the acid). However, this approximation will fail if the acid concentration is too low (<10^–6^ M) when autoprotolysis of water contributes significantly more to the total [H^+^] than the strong acid. For example, using this approximation, a solution of 10^–8^ M hydrochloric acid would have a pH of 8 at room temperature. This deviates significantly from the fact that a strong acid solution should not have a pH above 7 (the pH of a neutral solution). Such an example effectively shows undergraduates, typically at freshman and sophomore levels, the importance of solving complex chemical equilibrium problems with the systematic treatment approach, which considers the charge and mass balances of the relevant chemical species in addition to their chemical equilibrium constants.

The systematic treatment to calculate the pH of a highly diluted strong acid (HA) solution with the formal acid concentration of *F* (<10^–6^ M, hence the approximation that thermodynamic activities equal concentrations) starts by considering the dissociation of the strong acid (eq 1) and the autoprotolysis of water (eq 2) simultaneously and as the only two sources of H^+^ in the solution

1 $$HA\to H^{+}+A^{-}$$

2 $$H_{2}O⇋H^{+}+{OH}^{-}$$

Based on eqs 1 and 2, the following eqs 3–5 can be established

3 $$K_{w}=\left[H^{+}\right]\left[{OH}^{-}\right]$$

4 $$F=\left[A^{-}\right]$$

5 $$\left[H^{+}\right]=\left[{OH}^{-}\right]+\left[A^{-}\right]$$

Equation 3 is the equilibrium constant of the autoprotolysis of water. Equation 4 defines the formal concentration *F* of the strong acid. Equation 5 is the charge balance equation of the solution.

By using rearrangement and substitution to eliminate all variables other than [H^+^], the equations above will lead to the following quadratic eq 6

6 $${\left[H^{+}\right]}^{2}-F\left[H^{+}\right]-K_{w}=0$$

Using the quadratic formula for eq 6 gives two roots

7 $$R1=\frac{F+\sqrt{F^{2}+4K_{w}}}{2}={\left[H^{+}\right]}_{total}$$

8 $$R2=\frac{F-\sqrt{F^{2}+4K_{w}}}{2}$$

Note that the numerical value of *R*1 in eq 7 is always positive and that of *R*2 in eq 8 is always negative. The value of eq 7 gives the total H^+^ concentration of the solution, noted as [H^+^]_total_. *R*2 is normally discarded for the lack of physical meaning, as a concentration of a chemical species in an aqueous solution cannot be a negative value.

In addition, [H^+^] from the autoprotolysis of water (noted as [H^+^]_water_) can also be calculated from eqs 5 and 7, based on the following mass balance equation of H^+^ ^3^

9 $${\left[H^{+}\right]}_{total}={\left[H^{+}\right]}_{water}+{\left[H^{+}\right]}_{acid}$$

where the [H^+^]_total_ and [H^+^]_acid_ equal *R*1 in eq 7 and *F*, respectively. As a result, the concentration of H^+^ from the autoprotolysis of water should simply be

10 $${\left[H^{+}\right]}_{water}={\left[H^{+}\right]}_{total}-F=R1-F$$

As a numeric example, the pH of a solution with 10^–8^ M hydrochloric acid at room temperature (when *K*_w_ = 10^–14^) should be

11 $${\left[H^{+}\right]}_{total}=\frac{F+\sqrt{F^{2}+4K_{w}}}{2}=\frac{10^{-8}+\sqrt{10^{-16}+4\times 10^{-14}}}{2}=1.05\times 10^{-7}M$$

The negative root of this quadratic equation is calculated as −0.95 × 10^–7^ M. Furthermore, based on eq 10, the concentration of H^+^ from the autoprotolysis of water should be

12 $${\left[H^{+}\right]}_{water}={\left[H^{+}\right]}_{total}-F=1.05\times 10^{-7}Mto\;10^{-8}M=0.95\times 10^{-7}M$$

It appears that the absolute value of the negative root *R*2 is the same as that of the H^+^ concentration from the autoprotolysis of water. Is this a coincidence?

### Unexpected Application of the Negative Root

As it turns out, once thought to hold no significant value, the absolute value of the negative root *R*2 in eq 8 is *always* the same as [H^+^]_water_ because substituting eq 7 into 10 gives

13 $${\left[H^{+}\right]}_{water}=\frac{F+\sqrt{F^{2}+4K_{w}}}{2}-F=-\left(\frac{F-\sqrt{F^{2}+4K_{w}}}{2}\right)=-R2$$

Alternatively, the sum of the two roots of eq 6 is

14 $$R1+R2=\frac{F+\sqrt{F^{2}+4K_{w}}}{2}+\frac{F-\sqrt{F^{2}+4K_{w}}}{2}=F={\left[H^{+}\right]}_{acid}$$

Since ${\left[H^{+}\right]}_{total}=R1$ , combining eqs 9 and 14 gives the same conclusion that ${\left[H^{+}\right]}_{water}=-R2$ .

## Discussion and Conclusion

While a few articles^4–6^ have been published about the systematic treatment of chemical equilibria, to the best of our knowledge, this is the first time that the negative root of a quadratic equation from this approach shows a new meaning in solving for the pH contribution from the autoprotolysis of water. The proof in this article is based solely on the systematic treatment of equilibria without any other approximation beyond the ones on activities equaling concentrations and the absence of any other acid (such as CO_2_) in the system.

Such a revealed new meaning could be used in undergraduate analytical or physical chemistry curricula to highlight scenarios where the common assumption to neglect the autoprotolysis of water fails in pH calculations. Students may enhance their learning experience by practicing algebraic deductions in a chemistry problem involving a highly diluted strong acid or base solution. Either a problem-solving- or a discovery-based learning approach can be used.

After the systematic treatment of a highly diluted strong acid solution is discussed in the lecture, the instructor can present the fact that the absolute value of the negative root and [H^+^]_water_ are numerically the same and offer an in-class or homework problem for students to explain how they are algebraically related to each other. The instructor could also present the proof for the highly diluted strong acid solution and lead the students to explore the [OH^–^] from autoprotolysis of water in a highly diluted strong base solution (such as the NaOH aqueous solution below 10^–6^ M). In this case, similar systematic treatment of equilibrium equations can be established. After proper rearrangement and substitution to eliminate all variables other than [OH^–^] in a quadratic equation, the total [OH^–^] can be solved as the positive root. Following the method presented in this article, it can be shown that the absolute value of the negative root equals [OH^–^] from the autoprotolysis of water.

Alternatively, the instructor can guide students to critically evaluate the common practice of ignoring autoprotolysis of water in aqueous pH calculations by examining the ratios of [H^+^]_total_ and [H^+^]_water_ across a wide range of acid concentrations (e.g., Table 1, calculated similarly to the numeric example in eqs 11 and 12). The students can numerically examine the values of [H^+^]_water_ and −*R*2 and notice that they are the same. The students can then be guided to establish some hypotheses on the possible reasons for this observation. The students can then be mentored to prove their hypotheses using the equations from the systematic treatment of such a chemical system.

**Table 1:** Calculated Total [H^+^], [H^+^] from Autoprotolysis of Water and Solution pH for Strong Acid Solutions with *F* between 10^–7^ and 10^–9^ M at Room Temperature

*F* (×10^–8^ M)	*R*1 (×10^–8^ M)	*R*2 (×10^–8^ M)	[H^+^]_total_ (=*R*1, × 10^–8^ M)	pH	[H^+^]_water_ (=*R*1 −*F*, × 10^–8^ M)
10.0	16.18	–6.18	16.18	6.791	6.18
7.50	14.43	–6.93	14.43	6.841	6.93
5.00	12.81	–7.81	12.81	6.893	7.81
2.50	11.33	–8.83	11.33	6.946	8.83
1.00	10.51	–9.51	10.51	6.987	9.51
0.100	10.05	–9.95	10.05	6.998	9.95

Finally, this article assumes that there is no dissolved CO_2_ in the solution for simplicity. However, undergraduate students could be encouraged to examine the system while considering the contribution of dissolved CO_2_ (perhaps with a wide range of concentrations) in the aqueous solution and further practicing their learned skills on the systematic treatment of chemical equilibria.

## †

Department of Mechanical Engineering and Applied Mechanics, University of Pennsylvania, Philadelphia, PA 19104
